# Hypoxia-selective inhibition of angiogenesis development by NAMI-A analogues

**DOI:** 10.1007/s10534-016-9974-9

**Published:** 2016-11-03

**Authors:** Maria Oszajca, Guillaume Collet, Grażyna Stochel, Claudine Kieda, Małgorzata Brindell

**Affiliations:** 1 Faculty of Chemistry, Jagiellonian University, Ingardena 3, 30-060 Kraków, Poland; 2 Centre for Molecular Biophysics, Cell Recognition and Glycobiology, UPR4301-CNRS, rue Charles Sadron, 45071 Orléans, France; 3 Malopolska Biotechnology Centre, Jagiellonian University, Kraków, Poland

**Keywords:** Ruthenium complexes, NAMI-A analogues, Angiogenesis, Hypoxia, Endothelial cells

## Abstract

The antimetastatic ruthenium(III) complex (H_2_Im)[*trans*-RuCl_4_(HIm)(DMSO)] (NAMI-A) as well as its two analogues (H_2_Ind)[*trans*-RuCl_4_(HInd)(DMSO)] (Ru-Ind) and (HIsq)[*trans*-RuCl_4_(Isq)(DMSO)] (Ru-Isq) (HIm–imidazole, HInd–indazole, Isq–isoquinoline, DMSO–dimethyl sulfoxide) were tested for their effect on endothelial cell functions in vitro on human skin microvascular endothelial cells (HSkMEC) and human endothelial progenitor cells (HPEC-CB.2) under normoxic (21 % O_2_) and hypoxic (1 % O_2_) conditions. All studied complexes showed very low cytotoxicity profiles towards both mature microvascular and precursor endothelial cells (ECs), independently of oxygen concentration. Among tested compounds Ru-Ind exhibited the highest cytotoxicity. The antiangiogenic activity of ruthenium complexes was evaluated for their influence on pseudo-vessels formation by microvascular endothelial cells (HSkMEC) because of their involvement in melanoma progression. Our studies indicated that Ru-Ind and Ru-Isq exhibited hypoxia- and dose-dependent-inhibition of angiogenesis on Matrigel™. Significant hypoxia-selective downregulation of pseudo-vessels formation by Ru-Isq correlates with efficient inhibition of cell motility. Interestingly, in the applied concentration doses migration of endothelial cells was also inhibited by NAMI-A, but the pseudo-vessels formation on Matrigel™ was unaffected. Angiogenesis-related genes expression profile for both mature and precursor ECs indicated that inhibition of angiogenesis, mainly due to Ru-Isq, as compared to NAMI-A and Ru-Ind correlated with downregulation of CD31 and CD144 expression and upregulation of NOTCH4 expression in mature ECs, which is essential for endothelial cell motility and stalk cells organization control. The hypoxia-selective antiangiogenic activity of Ru-Ind and Ru-Isq, NAMI-A analogues makes them potent antimetastatic therapeutics for their selective action in hypoxia which controls tumor pathologic angiogenesis.

**Electronic supplementary material:**

The online version of this article (doi:10.1007/s10534-016-9974-9) contains supplementary material, which is available to authorized users.

## Introduction

Angiogenesis is a crucial step for tumor growth, supplying tumor cells with oxygen, nutrients and allowing metastasis. Cancer tissues are characterized by hypoxic areas (areas with O_2_ tensions <10 mmHg) which arise from restricted oxygen supply due to inefficient blood vessel formation in fast growing neoplastic tissues. Hypoxia is a typical property of locally advanced solid tumors and stem cell niches. It has been estimated that 50–60 % of malignancies exhibit chronic hypoxic/anoxic areas (Vaupel and Mayer 2007). Secondary tumors often have even poorer oxygenation level than primary tumors (Höckel and Vaupel 2001). Therefore, it is of great importance to carry out in vitro experiments not only under normoxia but also under hypoxia conditions.

Tumor angiogenesis is a complex process of reaction to hypoxia and results in the formation of incomplete, leaky, permeable vessels, inefficiently supplying oxygen and nutrients to tumors cells (Kieda et al. 2013). Generally, the vicinal endothelial cells are recruited for progressing angiogenesis. However, there is more and more evidence that the mobilization of circulating endothelial progenitor like cells (EPCs) also takes place, resulting in improved neovascularization (Asahara et al. 1997). To follow this finding the evaluation of the effect of the studied compounds on angiogenesis was performed using human skin mature microvascular endothelial cell line (HSkMEC) (Kieda et al. 2002) and a human progenitor endothelial cell line (HPEC-CB.2) (Collet et al. 2016; Paprocka et al. 2011).

**Fig. 1:**
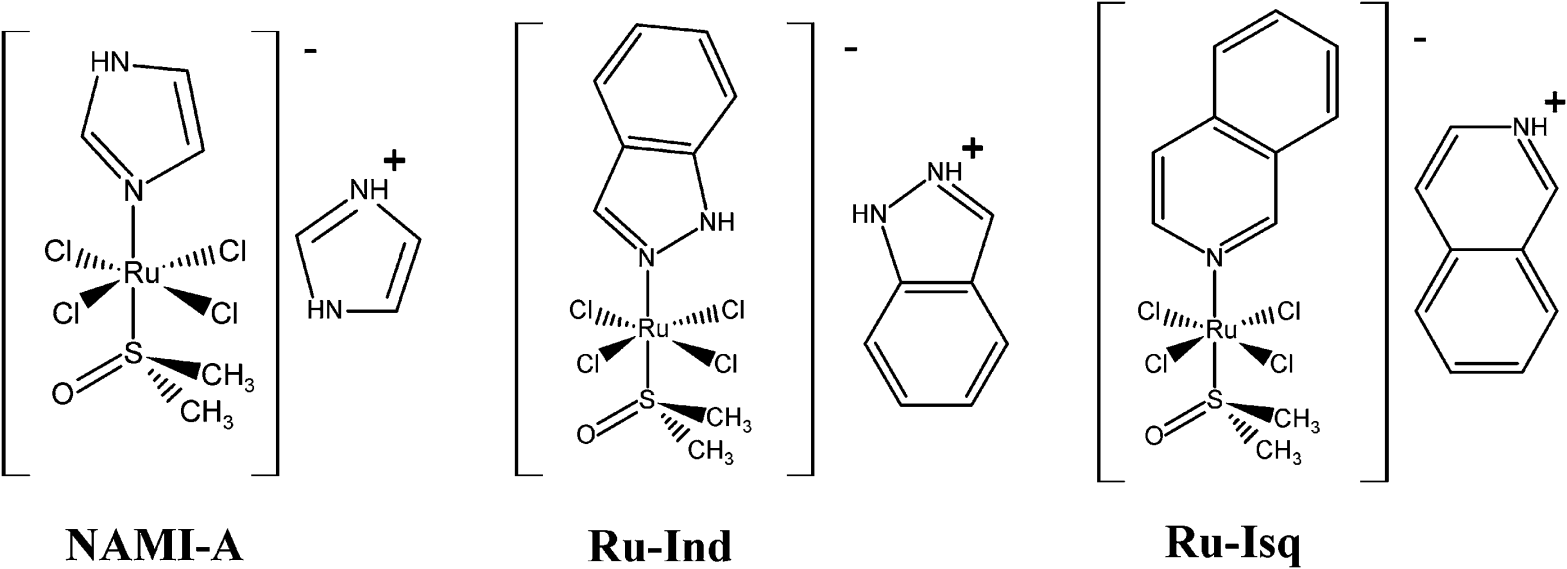
Complexes studied in this work NAMI-A, Ru-Ind and Ru-Isq

The aim of the present study was to assess the antiangiogenic activity of the antimetastatic ruthenium(III) complex (H_2_Im)[*trans*-RuCl_4_(HIm)(DMSO)] (NAMI-A) as well as its two analogues (H_2_Ind)[*trans*-RuCl_4_(HInd)(DMSO)] (Ru-Ind) and (HIsq)[*trans*-RuCl_4_(Isq)(DMSO)] (Ru-Isq) (HIm–imidazole, HInd–indazole, Isq–isoquinoline, DMSO–dimethyl sulfoxide) (Fig. 1) and decipher their role in biologically relevant conditions such as hypoxia, which exists in tumor proangiogenic sites. This also included comparing the activity on precursor endothelial cells with activity on mature endothelial cells that mimic the in vivo angiogenic process as well as angiogenesis control in tip cells with respect to stalk forming cells. To date only NAMI-A complex was evaluated in terms of its influence on angiogenesis in in vitro and in vivo studies (Bergamo and Sava 2015). It was found that NAMI-A can control angiogenesis most likely due to scavenging of NO produced by endothelial cells (ECs). The antiangiogenic effect of NAMI-A is related to inhibition of the MEK/ERK signaling pathway, downregulation of C-MYC gene expression and endothelial cell proliferation (Morbidelli et al. 2003; Pintus et al. 2002; Vacca et al. 2002).

In this study the impact of ruthenium complexes on angiogenesis is assessed by detection of pseudo-vessels formation as well as changes in cell migration of mature microvascular ECs. To investigate the induction and regulation of angiogenesis genes after the Ru complexes treatment, comparison of mRNA expression of a set of angiogenesis related genes for the ECs, both mature microvascular and precursor was performed, which allows to shed more light on the mechanisms of activity on these two types of cells. Importantly, we report for the first time the effect of NAMI-A and its two analogues (Ru-Ind and Ru-Isq) on angiogenesis of ECs under biologically relevant hypoxic conditions. Comparison of the results obtained in normoxia and hypoxia demonstrated the hypoxia-selective inhibition of angiogenesis by Ru-Ind and Ru-Isq. The revealed hypoxia-selective angiostatic properties of Ru-Ind and Ru-Isq makes these complexes candidates for further detailed investigation in view of medical applications.

## Materials and methods

### Ruthenium complexes

All chemicals used in this study were of analytical reagent grade. All three complexes (H_2_Im)[*trans*-RuCl_4_(HIm)(DMSO)], (H_2_Ind)[*trans*-RuCl_4_(HInd)(DMSO)] and (HIsq)[*trans*-RuCl_4_(Isq)(DMSO)] were prepared following the published procedure (Capozzi et al. 1998; Mestroni et al. 1998; Reisner et al. 2004), and their purity was confirmed by elemental analyses. (H_2_Im)[*trans*-RuCl_4_(HIm)(DMSO)] %: C, 20.97; H, 3.30; N, 12.23. Found, %: C, 21.31; H, 3.33; N, 12.28; (H_2_Ind)[*trans*-RuCl_4_(HInd)(DMSO)] %: C, 34.42; H, 3.43; N, 10.04. Found, %: C, 34.53; H, 3.42; N, 10.06; (HIsq)[*trans*-RuCl_4_(Isq)(DMSO)] %: C, 41.39; H, 3.65; N, 4.83. Found, %: C, 41.48; H, 3.61; N, 4.66. Ruthenium complexes were dissolved in water (except for (HIsq)[*trans*-RuCl_4_(Isq)(DMSO)]) and diluted to appropriate concentration in medium without serum or in complete medium with 2 % serum immediately before use. (HIsq)[*trans*-RuCl_4_(Isq)(DMSO)] was dissolved in DMSO due to poor water solubility and diluted in medium without serum and in complete medium with 2 % serum (final DMSO concentration was kept constant at 0.5 % v/v for cytotoxicity study and 0.1 % v/v for all other experiments) just before cells treatment.

### Cell lines

Two immortalized cell lines were used in this study: HSkMEC (Kieda et al. 2002)–human skin microvascular endothelial cells and HPEC-CB.2–human progenitor endothelial cells (Collet et al. 2016; Paprocka et al. 2011). Cells were cultured in OptiMEM with Glutamax (Invitrogen, Cergy Pontoise, France), supplemented with: 0.4 % (w/v) gentamicin (Invitrogen), 0.2 % fungizone (Invitrogen), 2 % (v/v) fetal bovine serum (BioWest, Nuaille, France). Cells were routinely maintained at 37 °C in a humidified incubator in a 5 % CO_2_/95 % air atmosphere.

### In vitro cytotoxicity evaluation

Cells (HSkMEC and HPEC-CB.2) were seeded (1 × 10^4^ cells per cm^2^) on 96-well cell culture plates in culture medium and grown for 24 h in a humidified incubator in a 5 % CO_2_/95 % air atmosphere at 37 °C. Cells were then incubated for 24 and 48 h in normoxic or hypoxic conditions with the tested compounds in medium without serum and in complete medium with 2 % serum. For hypoxia treatment, cells were placed in a humidified atmosphere containing 1 % O_2_, 94 % N_2_ and 5 % CO_2_ gas mixture, 37 °C in a hypoxic station Whitley H35. For the treatment at hypoxic conditions, medium was conditioned at hypoxic chamber for 24 h before use. At the end of incubation time, ruthenium compound solutions were removed and replaced with Alamar Blue™ dye (Biosource, Nivelles, Belgium) and incubated for 3 h at 37 °C. Fluorescence was measured at 605 nm (excitation wavelength 560 nm) with the application of VICTOR 3 V multilabel plate reader from PerkinElmer.

### Angiogenesis assay

Cells (HSkMEC) were seeded at the density of 2 × 10^4^ cells per cm^2^ in a complete medium with 2 % of serum. The studied compounds prepared in a medium without serum were added to the cell cultures 24 h after seeding and were later incubated for 24 h in normoxic and hypoxic conditions. Angiogenesis assay was performed on 96-well plates coated with Matrigel™ (BD Biosciences, Grenoble, France). Cells were treated with trypsin and seeded 5 × 10^4^ cells per cm^2^ in the presence of the tested compounds in medium without serum. The real-time visualization of the pseudo vessels formation was monitored during 24 h under normoxic and hypoxic conditions (1 % O_2_, 94 % N_2_ and 5 % CO_2_ gas mixture, 37 °C). The AxioVert 200 M videomicroscope (Carl Zeiss) equipped with temperature, gas and humidity controllers was used to acquire images in time lapse conditions. Angiogenesis was evaluated based on the formation of pseudo-vessels on Matrigel™.

### Migration assay

Cells (HSkMEC) were seeded at 2 × 10^4^ cells per cm^2^ in a complete medium with 2 % of serum. The influence of complexes on cells motility was tested on uncoated, plasma treated plastic plates. The studied compounds prepared in a medium without serum were added to the cell cultures 24 h after their seeding and incubation was allowed for 24 h in normoxic and hypoxic conditions. Confluent cell surface was scratched with 200 μl tip. Culture medium was removed and replaced by fresh medium containing the studied compounds at the dose of 100 μM. Images were captured using the AxioVert 200 M microscope (Carl Zeiss) at the beginning of the experiment and at regular intervals during cell migration to close the scratch.

### Influence of the studied compounds on gene expression

Cells (HSkMEC, HPEC-BC.2) were seeded at 2 × 10^4^ cells per cm^2^ in a complete medium with 2 % of serum. The studied compounds prepared in medium without serum were added to cell cultures 24 h after seeding followed by 24 h incubation in normoxic and hypoxic conditions. mRNA was extracted from these cells using RNeasy Plus Mini kit (Qiagen). mRNA concentration and quality was checked using NanoDrop spectrophotometer ND-1000 (NanoDrop Technologies, Wilmington, DE, USA). Reverse transcription was performed with the application of “Maxima First Strand cDNA Synthesis Kit” (Fermentas). Real-time PCR was performed using SYBRGreen Premix Ex Taq (Takara Bio Inc.). HPRT1 was used as housekeeping gene (HKG).

### Statistical analysis

Reported values are given as mean ± SD. Data are represented as averages of three biological replicates, performed in duplicate or triplicate. Statistical analyses were performed using the Student’s t test with p < 0.05 considered statistically significant.

## Results and discussion

### Cytotoxicity assay of Ru complexes

**Fig. 2:**
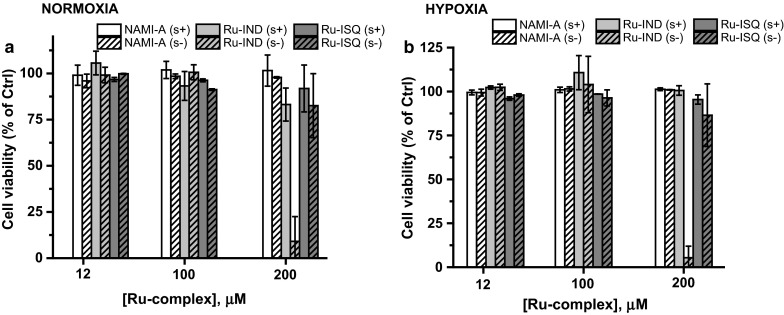
Cytotoxic effect of studied compounds on HSkMEC under normoxic (**a**) and hypoxic (**b**) conditions, NAMI-A–(H_2_Im)[*trans*-RuCl_4_(HIm)(DMSO)], Ru-Isq–(HIsq)[*trans*-RuCl_4_(Isq)(DMSO)], Ru-Ind–(H_2_Ind)[*trans*-RuCl_4_(HInd)(DMSO)] in medium with serum (s+) and without serum (s−) after 24 h of incubation. Values are mean ± SD of the three independent experiments

Cytotoxicity of the studied ruthenium complexes was assessed under normoxic and hypoxic conditions after 24 and 48 h of incubation. The studied ruthenium complexes were screened for their ability to reduce viability of two endothelial cell lines: HSkMEC and HPEC-CB.2. In the applied concentration range (1.5–200 μM) for each of the compounds only Ru-Ind reduced HSkMEC viability at a concentration higher than 100 μM. This occurred both in normoxia and hypoxia when compound was administered in medium without serum (Figs. 2, Suplementary Material Fig. S1). In medium with serum, the effect on endothelial cells was slightly less pronounced under both normoxic and hypoxic conditions (Suplementary Material Fig. S1). Two other ruthenium complexes, NAMI-A and Ru-Isq did not inhibit the growth of HSkMEC at dose up to 200 μM (Figs. 2, Suplementary Material Fig. S1). However, it was shown that in human umbilical vein endothelial cells (HUVEC), which are commonly used but are macrovascular ECs, the NAMI-A complex becomes cytotoxic in concentration above 100 μM (Vacca et al. 2002), indicating cell-type specific effects of this compound.

A very similar cytotoxicity profile was obtained for HPEC-CB.2 cell line. Analogically, NAMI-A showed no cytotoxic effect up to 200 μM concentration, whereas only slightly pronounced effect of Ru-ISQ was observed for the highest studied dose, 200 μM, under normoxic and hypoxic conditions (Suplementary Material Fig. S2). Ru-Ind showed the strongest viability inhibition, with IC_50_ between 100 and 200 μM (Suplementary Material Fig. S2). Comparison of cytotoxicity profiles indicates a slightly higher sensitivity of HPEC-CB.2 than HSkMEC to the studied ruthenium complexes.

The obtained results revealed that, neither duration of the treatment nor oxygen availability (hypoxia vs. normoxia) had a significant influence on their viability in the presence of the ruthenium complexes. However, a meaningful difference was observed between experiments in medium with and without serum for cytotoxic doses of Ru-Ind. Thus the decreased activity of the complex may arise from the formation of ruthenium-protein adducts (Bergamo et al. 2003; Mazuryk et al. 2012; Timerbaev et al. 2006).

The observed higher cytotoxicity of Ru-Ind than NAMI-A in endothelial cell lines is in agreement with cytotoxicity profile for these complexes reported by Groessl et al. (2007) showing rather moderate cytotoxicity of these two ruthenium complexes, with IC_50_ above 170 µM in human tumor cell lines. Higher sensitivity of tumor cell lines to Ru-Ind than NAMI-A or Ru-Isq might arise from its hydrolytic behavior, which pointed to the complete dissociation of N-heterocyclic ligand under physiological like condition (Oszajca et al. 2016). In contrast, only ca. 30 % of isoquinoline dissociated from Ru-Isq, while no release of imidazole was observed for NAMI-A (Oszajca et al. 2016).

However, the observed cytotoxicity profile for Ru-Ind and Ru-Isq is in contrast to the results reported for very similar complexes bearing more lipophilic TMSO ligand instead of DMSO namely, Na[*trans*-RuCl_4_(Isq)(TMSO)] and Na[*trans*-RuCl_4_(HInd)(TMSO)] (TMSO–tetramethylsulfoxide). Capozzi et al. (1998) reported that Na[*trans*-RuCl_4_(Isq)(TMSO)] is much more cytotoxic than Na[*trans*-RuCl_4_(HInd)(TMSO)] on TLX5 lymphoma, comparably to cisplatin. This is in contrast with our results showing that Ru-Isq is devoid of any detectable cytotoxic effect on HSkMEC in the studied concentration range similar to the used concentration of NAMI-A, whereas Ru-Ind displayed a significant cytotoxic effect only at higher doses when administered in medium without serum. Much higher cytotoxic effect of Na[*trans*-RuCl_4_(Isq)(TMSO)] and Na[*trans*-RuCl_4_(HInd)(TMSO)] was observed on a cancer cell line, when compared with analogues studied by us on endothelial cells. This seems to be related to the presence of TMSO ligand which provides higher lipophilicity of the complexes. This in turn may favor a cellular uptake and result in a more significant effect on cell viability. Furthermore, these results also stress that the strength of coordination of N-heterocyclic ligand to ruthenium ion plays a major role in the cytotoxic effect of studied complexes.

The absence of a lethal effect of the studied Ru-Ind and Ru-Isq up to high doses (similarly to NAMI-A) on endothelial cell lines indicates the pharmacological potency of the selected ruthenium complexes and encourages further study of their influence on angiogenesis development.

### Angiogenesis assay

Since it has been already shown that antimetastatic activity of NAMI-A may be antiangiogenic-dependent (Morbidelli et al. 2003; Vacca et al. 2002), we decided to test antiangiogenic properties of the selected NAMI-A analogues and compare their activity with NAMI-A. The antiangiogenic activity of the ruthenium complexes was evaluated by the formation of pseudo-vessels on Matrigel™ by HSkMEC. The estimation of the influence of studied complexes on angiogenesis development by HPEC-CB.2 failed due to their limited capability to form angiogenetic network and a related inability to properly quantify the observed effect.

**Fig. 3:**
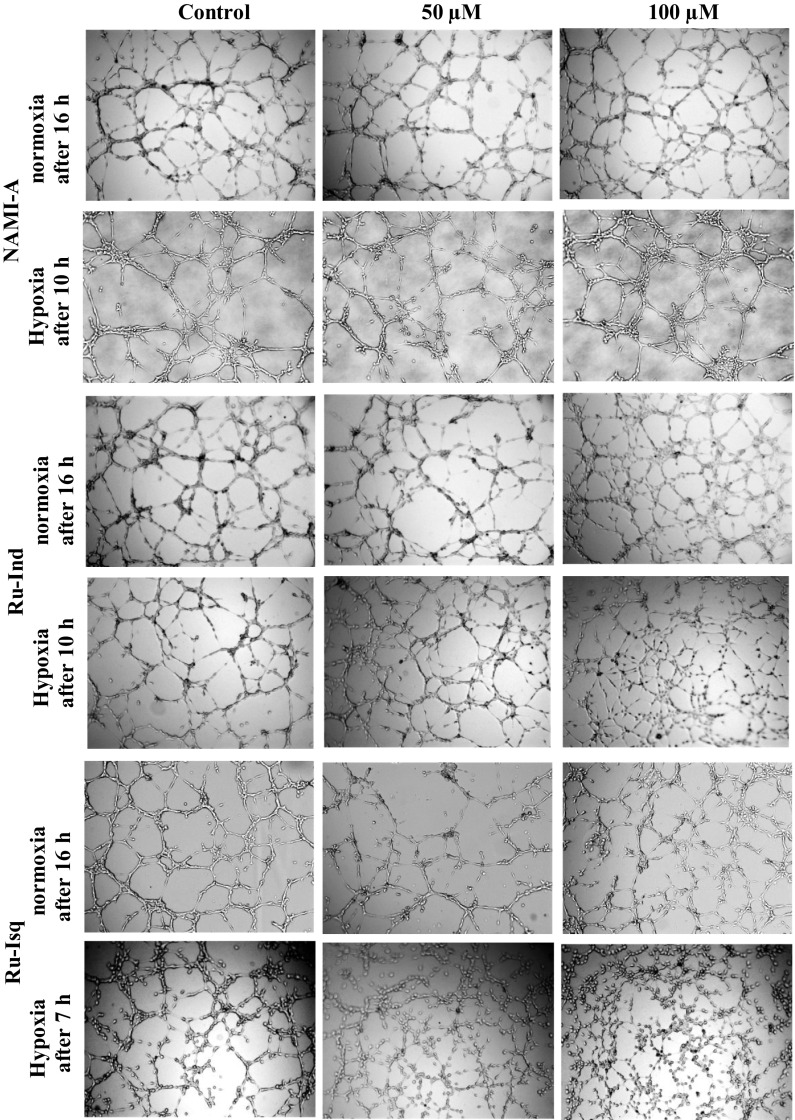
Effect of the ruthenium complexes on angiogenesis by skin endothelial cells (HSkMEC) under normoxic and hypoxic conditions. HSkMEC were pre-incubated in the presence of the ruthenium complexes (50 or 100 µM) for 24 h, then seeded on Matrigel™ in the presence of the same compounds

Since pseudo-vessel formation occurs much faster under hypoxic conditions, quantification of experimental data was performed after 7 h for hypoxic and 11 h for normoxic conditions. Influence of the studied ruthenium complexes on neovascularization was evaluated by comparison of two relevant descriptor variables: the number of formed pseudo-vessels and their mean length. Results of the representative data are shown in Table 1. Surprisingly, after the treatment with NAMI-A, only a slight effect on pseudo-vessel mean length was observed in both hypoxia and normoxia. This finding is in contrast to the results reported by Vacca et al. (2002) who showed a dose-dependent anti-angiogenic activity in the chorioallantoic membrane model. A more pronounced effect on angiogenesis development was observed after treatment with Ru-Ind and Ru-Isq, though hypoxic conditions significantly strengthened the anti-angiogenic activity when compared with normoxia. In normoxia only the pseudo-vessels mean length was suppressed in a dose-dependent manner, whereas the number of vessels was unaffected by ruthenium complexes (Table 1, see also Fig. 3). In contrast, both NAMI-A analogues exhibited concentration dependent diminishing of both vessel number and vessel mean length under hypoxic conditions. The inhibitory effect of Ru-Isq was slightly stronger than that of Ru-Ind (−76 vs. −56 % for vessel number at a dose of 100 µM, −28 vs. −35 % for vessel mean length at the same dose determined in relation to the control) (Table 1; Fig. 3). Very low cytotoxicity of the selected ruthenium complexes on HSkMEC indicates that the observed anti-angiogenic effect does not result from a cytostatic activity of Ru-Ind or Ru-Isq. The obtained results point to hypoxia-selective inhibition of angiogenesis development by Ru-Ind and Ru-Isq, which is a strongly desired property sought among ruthenium complexes studied as potential antimetastatic agents.

**Table 1:** Effect of the ruthenium complexes on HSkMEC angiogenesis

Cell treatment	Vessels number (>100 μM)	Vessels mean length (μM)		
		% Control	Mean ± SD	% Control
Normoxia				
Control	89	100	140 ± 67	100
[NAMI-A] = 50 μM	100	112	125 ± 65	89
[NAMI-A] = 100 μM	103	116	125 ± 64	89
Hypoxia				
Control	50	100	122 ± 69	100
[NAMI-A] = 50 μM	52	103	110 ± 55	90
[NAMI-A] = 100 μM	55	109	110 ± 56	90
Normoxia				
Control	101	100	136 ± 63	100
[Ru-Ind] = 50 μM	108	107	115 ± 45	84
[Ru-Ind] = 100 μM	104	103	86 ± 55	63
Hypoxia				
Control	79	100	109 ± 53	100
[Ru-Ind] = 50 μM	63	80	94 ± 44	87
[Ru-Ind] = 100 μM	35	44	71 ± 31	65
Normoxia				
Control/0.1 % DMSO	98	100	133 ± 65	100
[Ru-Isq] = 50 μM	137	139	122 ± 58	92
[Ru-Isq] = 100 μM	108	110	99 ± 44	74
Hypoxia				
Control/0.1 % DMSO	65	100	97 ± 43	100
[Ru-Isq] = 50 μM	31	48	79 ± 47	82
[Ru-Isq] = 100 μM	15	24	70 ± 25	72

Bergamo et al. (2002) reported that NAMI-A type complexes bearing less basic N-donor ligand than imidazole display higher antitumor activity, which was assigned to reduced DMSO loss. This conclusion seems to be in line with much higher effectiveness of angiogenesis development inhibition by Ru-Ind and Ru-Isq than NAMI-A that we observed. However, comparing basicity of N-heterocyclic ligands: indazole (p*K*_a_ = 1.25) vs. isoquinoline (p*K*_a_ = 5.40) one could expect higher activity of Ru-Ind than Ru-Isq, whereas our results pointed to Ru-Isq as the most active towards angiogenesis downregulation among the studied complexes. This implies that other factors arising from different hydrolytic behavior of both compounds need to be considered (Oszajca et al. 2016).

### Migration assay

**Fig. 4:**
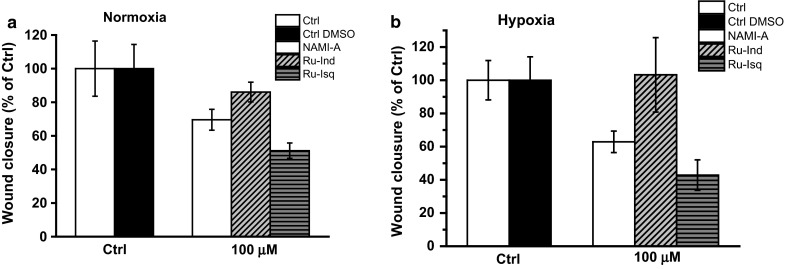
Effect of the studied ruthenium complexes on skin endothelial cells (HSkMEC) motility under normoxic (**a**) and hypoxic (**b**) conditions. Quantification was done after 12 h. Values are mean ± SD

As a second indication of activity of the ruthenium complexes on endothelial cell function, migration assays were performed using HSkMEC cells. Influence of the studied complexes on cells migration was determined 12 h after scratching and 36 h after exposure to the compounds at a dose of 100 μg/ml. Since cellular viability was not affected at such dose after 48 h incubation with all the studied complexes, the observed inhibition of migration is likely to result from an anti-angiogenic activity. Among the studied complexes two of them, namely NAMI-A and Ru-Isq, significantly diminished the motility of the endothelial cells, both in normoxia and hypoxia (Fig. 4, see also Suplementary Material Fig. S3–time dependent effect of studied compounds on HSkMEC migration). Migration was ~50 % inhibited for Ru-Isq complex and slightly less (~35 %) for NAMI-A in both normoxia and hypoxia (Fig. 3), whereas Ru-Ind was much less active under normoxic and inactive under hypoxic conditions.

The stronger influence of Ru-Isq on cell migration than two other complexes is in agreement with angiogenesis data (Table 1) and correlates well with its impact on the selected gene expression discussed in the following paragraph. Interestingly, Ru-Ind which exhibited significant effect on pseudo-vessels formation on Matrigel™ did not affect motility of the endothelial cells on uncoated, plasma treated plastic surface. In turn NAMI-A, which did not affect angiogenesis development by skin endothelial cells on Matrigel™, revealed significant inhibiting effect on cells motility. The observed influence of the studied complexes on HSkMEC angiogenesis and motility suggests different mechanism for their activity, most probably related to the nature of the studied compounds (Oszajca et al. 2016).

### Influence of the studied compounds on gene expression

**Fig. 5:**
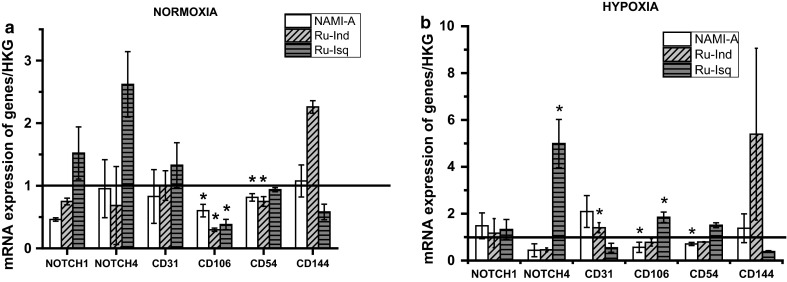
Influence of the ruthenium complexes on mRNA expression in HSkMEC in normoxia (**a**) and hypoxia (**b**). Values are mean ± SD of the three independent experiments, *p < 0.05

**Fig. 6:**
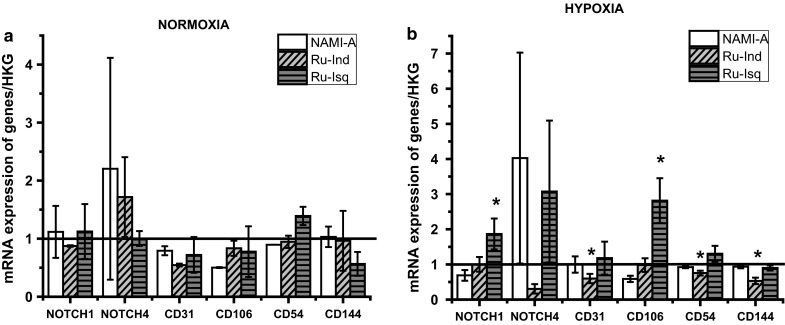
Influence of studied ruthenium complexes on mRNA expression in HPEC-CB.2 in normoxia (**a**) and hypoxia (**b**). Values are mean ± SD of the three independent experiments, *p < 0.05

To investigate the induction and regulation of angiogenesis genes after the treatment with ruthenium complexes, mRNA expression of a set of angiogenesis related genes for microvascular (HSkMEC) and progenitor (HPEC-BC.2) endothelial cell lines was determined. The expression of the following genes, with known implication in cell migration and pseudo-vessels formation, were analyzed: NOTCH1, NOTCH4, CD31/PECAM-1 (platelet endothelial cell adhesion molecule), CD106/VCAM-1 (vascular cell adhesion molecule 1), CD54/ICAM-1 (intercellular adhesion molecule 1), CD144/VE-cadherin (vascular endothelial cadherin). All obtained results are summarized in Figs. 5 and 6.

Among the studied complexes NAMI-A and Ru-Ind decrease the level of NOTCH1 and NOTCH4 in HSkMEC in normoxia, whereas under hypoxia only the level of NOTCH4 expression was diminished by these two complexes. In contrast, Ru-Isq increased level of NOTCH1 and NOTCH4 in normoxia and especially in hypoxia. NOTCH proteins are transmembrane receptors, which play a crucial role in cells fate, differentiation as well as angiogenesis development (Artavanis-Tsakonas et al. 1999; Dufraine et al. 2008; Greenwald 1998; Milner and Bigas 1999). Both NOTCH1 and NOTCH4 receptors are expressed in vascular endothelium and regulate vascular angiogenic remodeling (Krebs et al. 2000; Uyttendaele et al. 2001). NOTCH4 controls the proper vessel structure through inhibition of the tips cells progression in favor of the stalk structure. Leong et al. (2002) reported that constitutive NOTCH4 activation in human dermal microvascular endothelial cells inhibits angiogenesis in vivo and endothelial sprout formation* in vitro* both spontaneously as well as in response to FGF-2 and VEGF. The ability of NOTCH4 to inhibit angiogenesis was ascribed to induction of active conformation of β1-integrins, which results in promotion of adhesion (Leong et al. 2002). The significant increase in NOTCH4 expression level by Ru-Isq, especially in hypoxia is in agreement with the substantial inhibition of angiogenesis development by Ru-Isq on under hypoxia.

The influence of the ruthenium complexes on CD31 expression level in normoxia and hypoxia in HSkMEC is opposite. Under hypoxic conditions Ru-Isq diminished the level of CD31 expression, whereas under normoxic conditions this level increases. This is in contrast to the NAMI-A effect, which in hypoxia increased the expression level of this gene and slightly decreased it in normoxia. This is in agreement with the control of angiogenesis in hypoxia/normoxia as CD31 is important for vessels maturation. In the case of Ru-Ind no significant influence on CD31 expression in HSkMEC was observed. Interestingly, Ru-Isq also significantly diminished the expression level of the CD144 (vascular endothelial cadherin) in HSkMEC in hypoxia and normoxia. The observed decrease in the CD31 and CD144 expression levels caused by Ru-Isq, correlates with the observed destabilization of the HSkMEC pseudo-vessels network under hypoxic conditions. CD144 mediates homotypic cell adhesion and plays important role in vascular morphogenesis (Breviario et al. 1995). The significant role of CD31 in angiogenesis development has been confirmed by many studies. Carreau et al. (2011) have shown that downregulation of CD31 in HSkMEC expression correlates with the observed inhibition of pseudo-vessel formation by NO donors. Furthermore, earlier studies reported by Cao et al. (2002) proved that the increased expression of CD31 in cellular transfectants induced the tube formation and enhanced cell motility on Matrigel™. Significant contrasting effect on the CD144 expression level was also observed for Ru-Ind complex, since Ru-Ind significantly upregulated the expression of this gene in both normoxia and hypoxia. In contrast the expression level of CD144 after NAMI-A treatment remained almost unchanged. In normoxia all the studied complexes decreased the CD106 expression level in HSkMEC, whereas in hypoxia the same trend was observed only for NAMI-A and Ru-Ind. The influence of the studied ruthenium complexes on the CD54 expression level both in normoxia and hypoxia is generally similar. All of them slightly downregulate its expression except for Ru-Isq in hypoxia for which a slight increase in the expression of CD54 was observed.

In the case of HPEC-BC.2 no significant modulation of the NOTCH1 by studied complexes was observed in normoxia. Under hypoxia NAMI-A downregulated NOTCH1 expression, whereas Ru-Isq upregulated it. Significant increase in the expression level of NOTCH4 was determined for NAMI-A under normoxia and hypoxia. Furthermore, NAMI-A downregulated the expression level of CD31 in normoxia as well as CD106 under normoxia and hypoxia. Influence of Ru-Ind on expression level of NOTCH4 in normoxia and hypoxia is opposite. In normoxia, upregulation of NOTCH4 was observed after Ru-Ind treatment while downregulation was displayed in hypoxia. Ru-Ind also decreased expression levels of CD31 in normoxia and hypoxia, CD106 in normoxia, as well as CD54 and CD144 in hypoxia. Similarly to NAMI-A, Ru-Isq upregulated the expression of NOTCH4 in hypoxia, however it had no influence on this gene expression in normoxia. Significant upregulating effect of Ru-Isq was also observed on CD106 but only in hypoxia. Furthermore, Ru-Isq downregulated the expression of CD31, CD106, CD144 in normoxia and slightly upregulated the expression of CD54 in normoxia and hypoxia.

Altogether, these results suggest that significant angiogenesis inhibition by Ru-Isq, which is stronger than for the other studied ruthenium complexes may be due to the molecular mechanism which leads to decreasing CD31 and CD144 expression as well as increasing NOTCH4 expression, which appear essential for EC motility and organization of pseudo-vessels.

## Conclusions

This works brings a new view on the possibility to control angiogenesis. In this particular process it is of fundamental importance to design experimental conditions as close as possible to the biological ones. This is shown in particular by the clear differential effect exerted by the designed ruthenium complexes on the angiogenesis process in hypoxia as opposed to normoxia, as effects of the NAMI-A analogues are visible in hypoxia only.

The present work indicates that the properties of the complexes of interest could not have been evidenced in non-hypoxic conditions. This work also led to deciphering of the molecular mechanisms of the inhibitory effect on endothelial cells by pointing towards induction of NOTCH4 and its fundamental regulatory effect of the angiogenic process. Organospecificity of endothelium and the varied roles played by endothelial cells in the development of angiogenesis is reported here through the reaction of mature microvascular endothelial cells, which mimic in situ vessel sprouting during tumor development. The angiogenic properties of mature skin microvascular endothelial cells are inhibited by the action of Ru-Isq which was evidenced under hypoxic conditions. The second mechanism of tumor angiogenesis is recruitment of EPC mobilized from the bone marrow which was explored through the studying of the EPC line–HPEC.B2.

The use of NAMI-A analogues appears to be very promising as they are active towards the hypoxia-induced pathologic type of angiogenesis. Moreover, as their activity is exerted through NOTCH4 activation, these complexes may be able to control stalk formation and thus induce vessel normalization, which is a key mechanism to improve treatments and synergize chemotherapy and radiotherapy.

## Electronic supplementary material

Below is the link to the electronic supplementary material.

Supplementary material 1 (PDF 160 kb)
