# Supplementary material for: Hypoxia-selective inhibition of angiogenesis development by NAMI-A analogues
Source: Biometals. 2016 Nov 3;29(6):1035–46. doi: 10.1007/s10534-016-9974-9 (PMC5116311; doi:10.1007/s10534-016-9974-9)
Supplement: Supplementary file 1 — Supplementary material 1 (PDF 160 kb) [file 10534_2016_9974_MOESM1_ESM.pdf]

Supplementary Material

Selective-hypoxia inhibition of angiogenesis development by NAMI-A analogues

Maria Oszajca[a,*], Guillaume Collet[b], Grażyna Stochel[a], Claudine Kieda[b,c,*], Małgorzata Brindell[a]

[a] Faculty of Chemistry, Jagiellonian University, Ingardena 3, 30-060 Kraków, Poland

[b] Centre for Molecular Biophysics, Cell Recognition and Glycobiology, UPR4301-CNRS, rue Charles Sadron, Orléans 45071, France

[c] Malopolska Biotechnology Centre, Jagiellonian University, Kraków, Poland

***Corresponding author e-mail address: maria.oszajca@chemia.uj.edu.pl, claudine.kieda@cnrs-orleans.fr

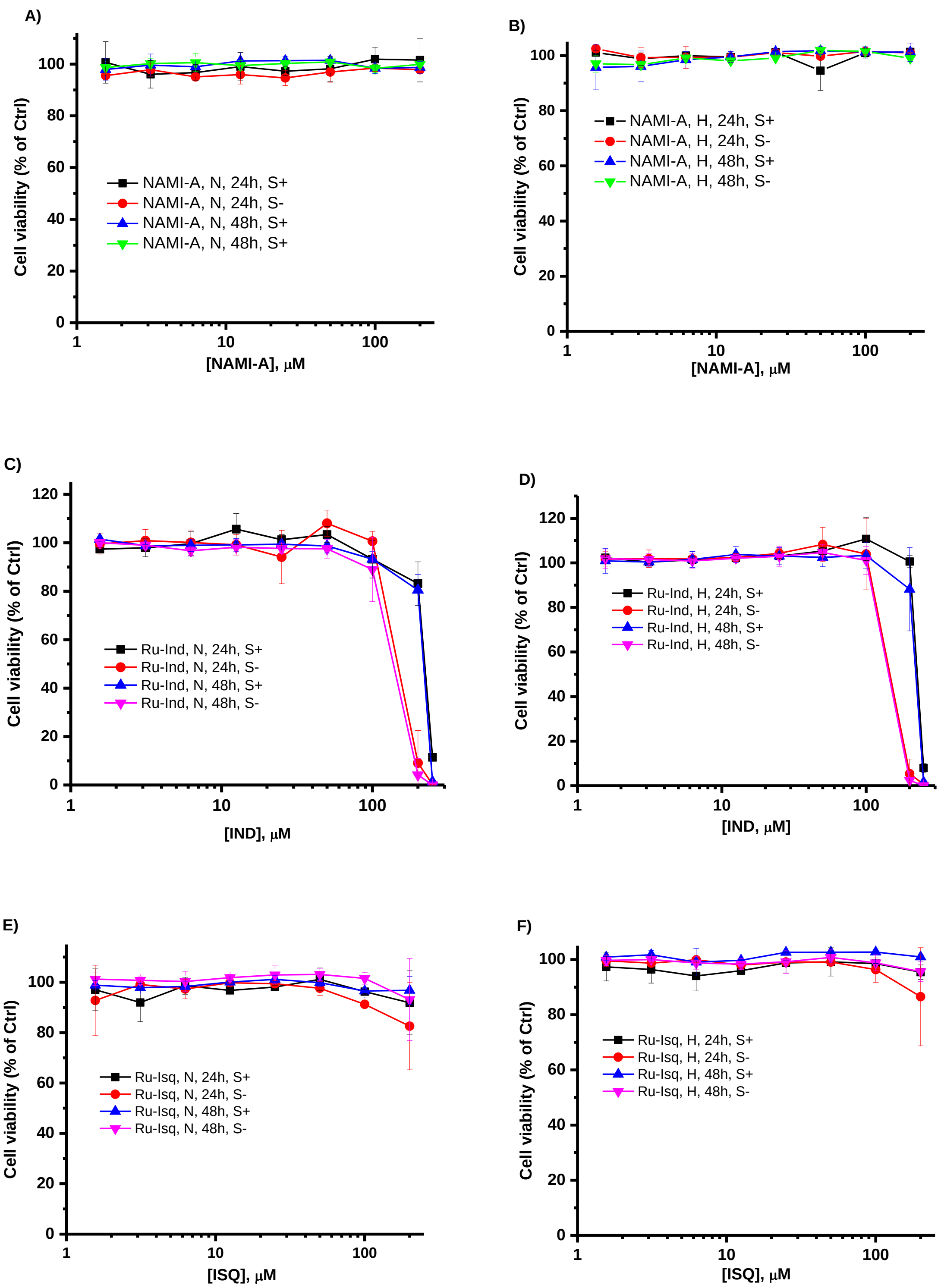

Fig. S1. Cytotoxic effect of studied compounds on HSkEC in A), C) and E) normoxic (N) and B), D) and F) hypoxic (H) conditions, A), B) - NAMI-A - [H_2Im][*trans*-$RuCl_4$(HIm)(DMSO)], C), D) - Ru-Ind - [H_2Ind][*trans*-$RuCl_4$(HInd)(DMSO)], E), F) - Ru-Isq - [HIsq][*trans*-$RuCl_4$(Isq)(DMSO)] in medium with serum (S+) and without serum (S-) after 24 h or 48 h of incubation.

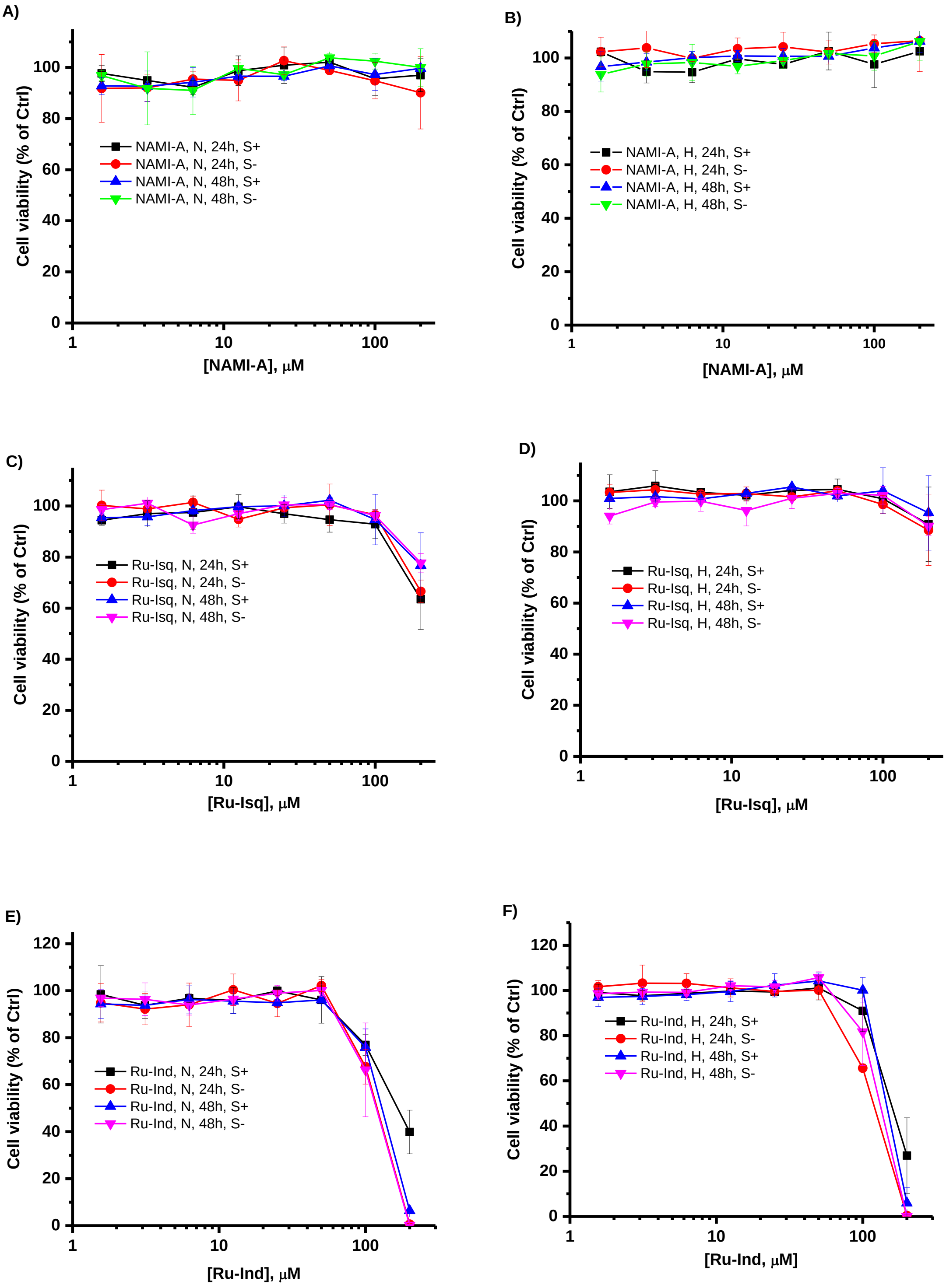

Fig S2. Cytotoxic effect of studied compounds on HPEC-CB.2. in A), C) and E) normoxic (N) and B), D) and F) hypoxic (H) conditions, A), B) - NAMI-A - [H_2Im][*trans*-$RuCl_4$(HIm)(DMSO)], C), D) - Ru-Isq - [HIsq][*trans*-$RuCl_4$(Isq)(DMSO)], E), F) - Ru-Ind - [H_2Ind][*trans*-$RuCl_4$(HInd)(DMSO)] in medium with serum (S+) and without serum (S-) after 24 h or 48 h of incubation.

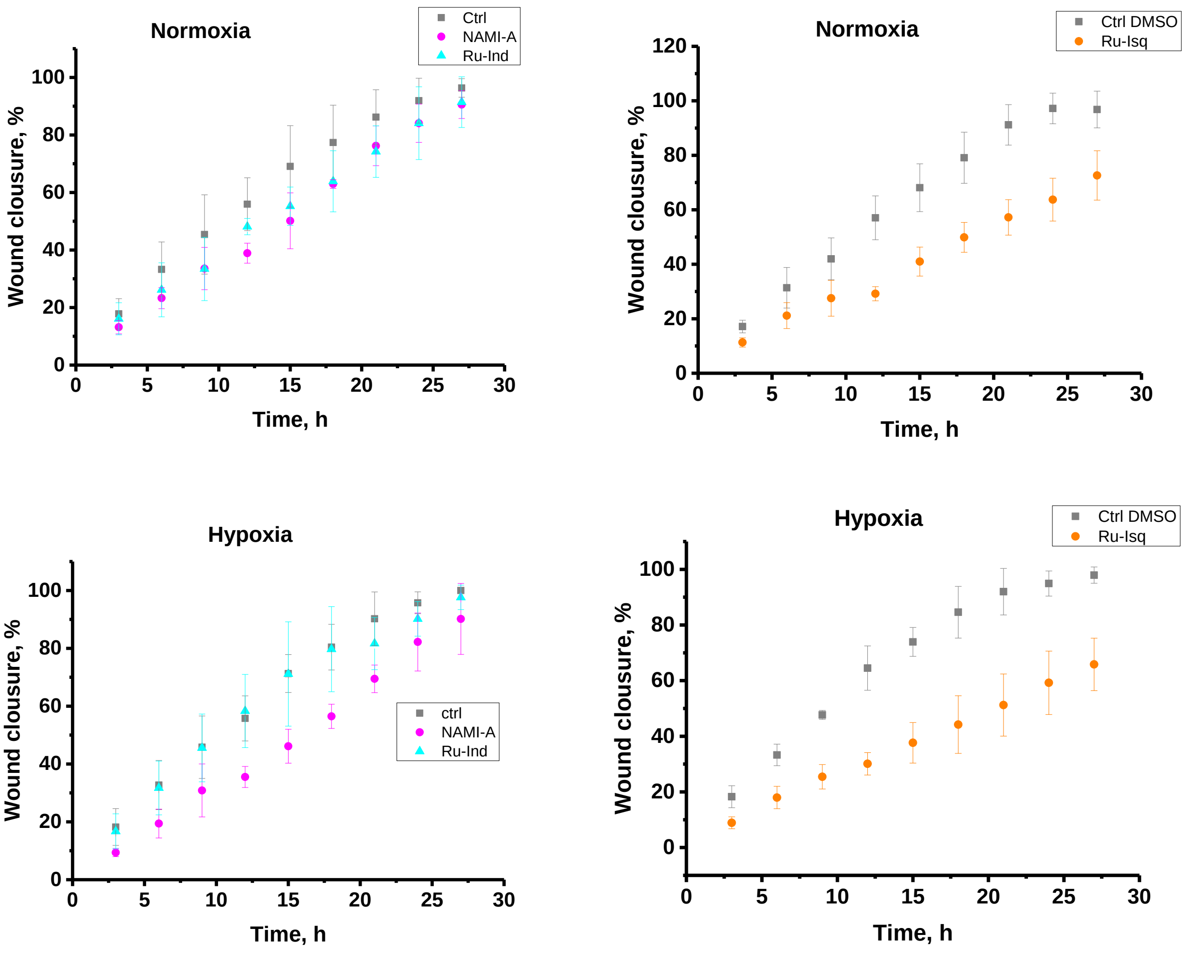

Fig S3. Time dependent effect of studied compounds on HSkMEC migration representing by time *vs*. confluence function curves. A), C) - NAMI-A - [H_2Im][*trans*-$RuCl_4$(HIm)(DMSO)], and Ru-Ind - [H_2Ind][*trans*-$RuCl_4$(HInd)(DMSO)]; B), D) - Ru-Isq - [HIsq][*trans*-$RuCl_4$(Isq)(DMSO)].
